# Sustainable Use of Fruit and Vegetable By-Products to Enhance Food Packaging Performance

**DOI:** 10.3390/foods9070857

**Published:** 2020-06-30

**Authors:** Flavia Dilucia, Valentina Lacivita, Amalia Conte, Matteo A. Del Nobile

**Affiliations:** Department of Agricultural Sciences, Food and Environment, University of Foggia, Via Napoli, 25-71121 Foggia, Italy; flavia_dilucia.552005@unifg.it (F.D.); valentina.lacivita@unifg.it (V.L.); matteo.delnobile@unifg.it (M.A.D.N.)

**Keywords:** fruit by-products, vegetable by-products, waste, active packaging, bioactive compounds, food chain sustainability

## Abstract

Fruit and vegetable by-products are the most abundant food waste. Industrial processes such as oil, juice, wine or sugar production greatly contribute to this amount. These kinds of residues are generally thrown away in form of leftover and used as feed or composted, but they are a great source of bioactive compounds like polyphenols, vitamins or minerals. The amount of residue with potential utilization after processing has been estimated in millions of tons every year. For this reason, many researchers all around the world are making great efforts to valorize and reuse these valuable resources. Of greatest importance is the by-product potential to enhance the properties of packaging intended for food applications. Therefore, this overview collects the most recent researches dealing with fruit and vegetable by-products used to enhance physical, mechanical, antioxidant and antimicrobial properties of packaging systems. Recent advances on synthetic or bio-based films enriched with by-product components are extensively reviewed, with an emphasis on the role that by-product extracts can play in food packaging materials.

## 1. Introduction

Every year there is a constantly growing rate of food lost and wasted all around the globe. According to FAO analysis [1], 1.3 billion of food are wasted annually. Food waste has been defined as *“food losses of quality and quantity through the process of the supply chain taking place at production, post-harvest and processing stages”* [2]. It is fundamental to understand that loss of food along the supply chain means loss of important resources like water, land and energy. Quantitatively, food waste costs for disposal and treatment, around US $680 billion in industrialized countries and US $310 billion in developing countries [3]. The management of the waste has become a global burden for governments. Generally, this kind of residues has been brought to the landfills and incinerated, and thanks to the affordability to face up this situation, this method of disposal and treatment has always been the simplest choice for municipalities [4,5]. As a result, year after year, there has been a reduction in land available for sending wastes and a growth of environmental concerns, like leaching generation and environmental pollution in terms of increasing biogas in the atmosphere and uncontrolled release of methane, all aspects guilty of the climate change of the planet.

The most abundant waste is represented by fruit and vegetable by-products, including roots and tubers, with a percentage of the residues around 40–50% of the total discards. By-products from fruit and vegetables are discarded in form of leftovers like seed, pulp, skin or pomace, accounting to 10–35% of raw mass [6,7,8]. Industrial processes are the main cause of these by-products, for example, grape and olive pomace derived from wine and oil production, other fruit by-products (apples, pears, peaches, citrus fruits, blueberries, mangoes, etc.) coming from juice, jelly and jam industries and also all waste from the processing of vegetables such as potatoes, tomatoes, fennels, artichokes or carrots [9]. Generally, they are used as animal feed or for production of biomaterials, biofuels, biogas, platform chemicals and bio-fertilizers through biological processes like fermentation or bio-electrogenesis [10,11]. By-products can also be used as substrate for production of lactic acid, a product that has applications in food, chemical and pharmachemical industries and can give origin to the PLA (PolyLactic Acid polymer) [12]. A particular use of agro-industrial by-products is the microbial processing application, in which several microorganisms can be used to transform the waste into new bio-product [13,14]. Examples are *Aspergillus sp.*, which can produce organic acid from by-products, or *Bacillus sp.*, particularly used for producing enzymes like cellulase, protease and amylase [15].

In general, food by-products have a huge potential of being recycled because are natural source of bioactive compounds such as simple sugars (glucose and fructose); carbohydrates; polysaccharides; pectin; fibers and valuable bioactive molecules like phenolic acids, carotenoids, tocopherols, flavonoids, vitamins and aromatic compounds. These compounds are very useful to human nutrition due to their antioxidant and antiviral properties [13,16].

**Figure 1:**
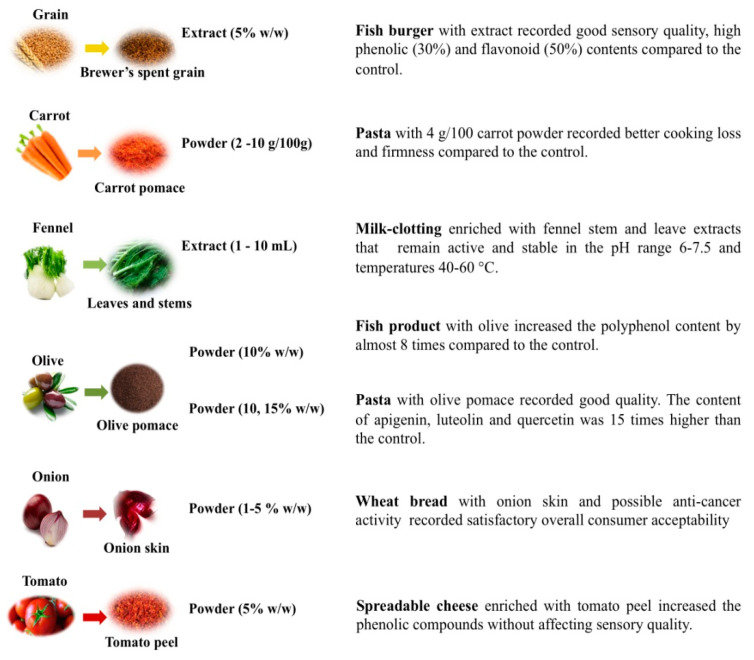
Applications of vegetable by-products as food ingredients.

**Figure 2:**
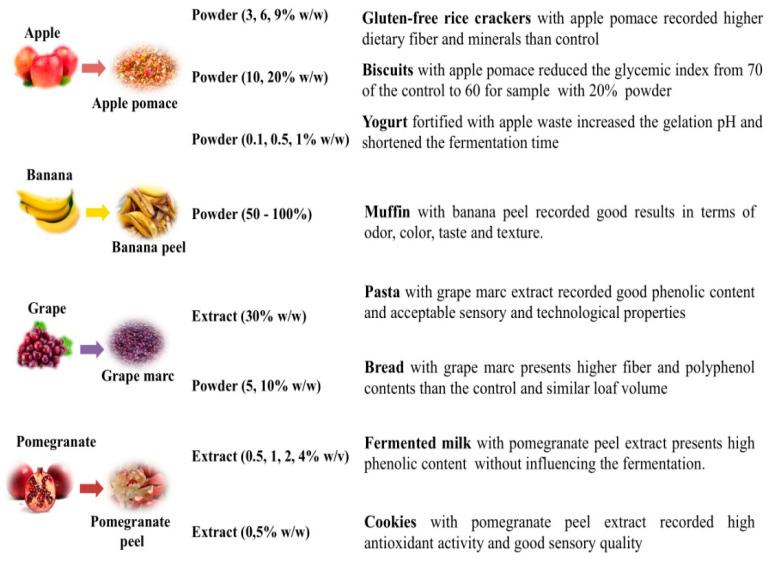
Applications of fruit by-products as food ingredients.

In fact, over the past few years, various researchers focused their attention on fruit and vegetable by-products as food ingredients. Figure 1 highlights some applications of vegetable by-products in form of powder or extract to fortify cereal-based [17,18,19], fish or dairy products [20,21,22,23]. Figure 2 reports other examples of food fortification by fruit by-products. Moreover, in this case, cereal-based [24,25,26,27,28,29] and dairy foods [30,31] have been enriched with powder or extract from these food residues to improve nutritional and technological properties.

As a feasible alternative to reduce the production cost of edible films and to add value to food by-products, the literature also reports the use of fruit and vegetable processing waste to develop edible films or coatings [32]. However, when the by-product is rich in components, oils or pigments with recognized properties, it makes sense to extract the compound of interest and add them to the film forming solution to improve final film properties [33,34,35,36]. The mechanical properties (tensile strength, flexibility, elongation at break, etc.) allow determining the strength of the material for food applications. Furthermore, the ability to absorb water and water vapor and gas permeability are important properties for food packaging. Plastics with low water absorption capacity and high barriers against moisture and gas are generally preferred. In addition, for perishable products, packaging with antioxidant and antimicrobial properties could represent a good opportunity to prolong the shelf life. Generally speaking, the incorporation of compounds of natural origin into the polymeric matrix, instead of synthetic additives, is more appreciated by consumers [37,38]. Therefore, due to the high amount of by-products that fruit and vegetable processing generates, their re-utilization in food packaging is expected to significantly contribute to develop new competitiveness, also considering the value of the global plastic food packaging market. 

Based on the above considerations, the aim of this review is to collect the most recent research about potential reuse and application of fruit and vegetable industrial residues to add value to both by-products and food packaging. Among the various components, particular attention will be paid to the role of by-product extracts for improving film performance.

## 2. Materials and Methods

For this review, electronic literature searches were conducted, including PubMed, Google Scholar and Science Direct databases. Numerous search terms have been used, including fruit by-products, vegetable by-products, active food packaging, antioxidant packaging, antimicrobial packaging. The research process uncovered numerous articles, and among them, 109 articles were used in the current overview. Literature data focused on the potential re-utilization of fruit and vegetable by-products, and how these resources can be used to improve physical, mechanical, antioxidant and antimicrobial properties of polymeric systems.

## 3. Fruit and Vegetable By-Products to Enhance Antioxidant Properties of Polymeric Film 

As reported above, fruit and vegetable by-products are very rich in phenolic compounds. Numerous researches deal with addition of skin, peel, seeds, pomace, kernel, leaves or their extracts to packaging to enhance the antioxidant properties (Table 1). Some researches are specifically discussed in the subsequent paragraphs dealing with fruit and olive oil industrial by-products. 

### 3.1. Fruit By-Products

Apple by-products represent 25–30% of the weight of the original fresh fruit. These residues are an important source of phenolic compounds, like hydroxycinnamic acid derivates and flavonoids [39,40,41]. According to the experimental findings of Urbina et al. [39], apple by-products could be used in a completely renewable active packaging. Specifically, the authors developed a bacterial cellulose-based nano-papers then coated with a medium-chain-length poly-hydroxy-alkanoate layer (mcl-PHA). Subsequently, extracts from cider by-products were incorporated at different percentages (1%, 3% and 5% respect to the PHA) to develop an antioxidant film with optimal free radical scavenging capacity.

As regard grape by-products, around 20% of whole grapes are generally rejected after the wine making process, even though these by-products are very rich in flavonoids and phenols (more that 70% are found in the skin) [50,51,52,53,54]. Interesting results were found in the study of Kurek et al. [50] who used two different bio-resources (red grape skin pomace and blueberry extracts) to develop a new active packaging with antioxidant properties. The extracts obtained by microwave-assisted extraction were incorporated into chitosan and carboxymethyl cellulose film, respectively. The films added with red grape skin extract exerted greater antioxidant activity than those enriched with blueberry extract, although an inverse correlation of polyphenol content and antioxidant activity was highlighted. Shahbazi [51] studied the potential application of grape seed extract (GSE 1% *w/v*) alone and in combination with an essential oil (ZEO, *Ziziphora clinopodioides*) in chitosan and gelatin films. The author observed that, in both films, the highest total phenolic content was found with the combination of both active compounds. Ferreira et al. [52] evaluated the effect of three grape pomace extracts (water extract 0.15%, wax extract 0.15–0.3% and oil extract 0.3–0.75%) into chitosan film. The study showed that the different nature of the extracts improved the antioxidant activity, also influencing the final film properties. Specifically, the wax extracts allowed obtaining films with better mechanical properties, whereas the lipid extracts reduced the solubility in water and led to slight change in the mechanical properties.

Mango by-products (kernel and peel) were also used in food packaging. Mango is the third most exported exotic fruit and its by-products are generally used for bio-refineries. Every year, about one million tons of mango seeds are eliminated by the industries without any other application, knowing that the seeds make up 20–60% of the whole fruit. An excellent use is reported in the study conducted by Melo et al. [55]. The researchers used three chemical fractions extracted from mango kernels (kernel starch, kernel fat and kernel phenolic extract) to develop active films. The authors highlighted that active systems exerted antioxidant capacity, UV absorbing and good barrier properties but reduced tensile properties and transparency. The possibility of conferring antioxidant activity to packaging by adding mango kernel extract, was also studied by Adilah et al. [56]. They observed that the incorporation of the extract into two biopolymers (soy protein isolate and fish gelatin) improved the film antioxidant activity. The authors justified the better results of the soy protein-based film with the globular structure of the raw material and the likely lower protein-phenol interaction compared to the linear structure of fish gelatin film. In a more recent study Adilah et al. [57] assessed the stability of a soy protein-based film enriched with the same mango kernel extract during 90 days of storage at different temperatures (25, 4 and −18 °C). The total phenolic content of film stored at room temperature was around 26 to 50% higher than films at 4 and −18 °C, respectively. Regarding the antioxidant activity, it remained stable at 25 °C, while it slight decreased at refrigeration and freezing temperature. These results can be associated not only to the loss of extract during storage but also to possible change in the film protein structure. Mango leaf extract was less explored, even if it is rich in gallic acid, glucosides and phenolic compounds [58]. The study conducted by Rambabu et al. [59] evaluated the incorporation of mango leave extract (1%, 3% and 5%) into chitosan, and then the application of the active system to cashews. As expected, the total phenolic content of films increased as the extract increased. Peroxide values monitored for 28 days highlighted that 3% and 5% enriched films better inhibited the oxidation process of cashews.

Pomegranate by-products, such as peel, seed and pomace, were re-used as derivates from pomegranate juice and jam industry. High scavenging properties of free radicals, antimicrobial and anti-mutagenic properties of pomegranate peel extract increased the interest in this by-product, thus leading to develop active films [65,66,67,68]. Hanani et al. [66] used pomegranate, papaya and jackfruit peel in form of powder to develop a bi-layer film made up of fish gelatin and polyethylene (PE). In particular, the three by-products were added (0–9% *w/v*) separately to the fish gelatin solution, and then, each active film was cast onto the PE layer to form a bi-layer matrix. The comparison among the three active kinds of packaging showed that pomegranate peel powder was the most interesting by-product because it has a high phenolic content and gave the films a good antioxidant activity. These results were also confirmed in a subsequent study [67] where, together with antioxidant properties deriving from the addition of pomegranate peel powder, the barrier properties of active films were also studied.

### 3.2. Olive-Oil Industrial By-Products

The olive oil industrial production process generates a huge quantity of olive residues generally discarded, even if these by-products are very rich in dietary fibers, phenolic compounds and carotenoids [60,61,62,63,64]. Olive pomace flour (10%, 20% and 30% in relation to the mass of chitosan) and microparticles prepared by spray drying (10%, 20% and 30%) were incorporated into chitosan films [60]. The antioxidant properties of olive pomace flour was higher than that of microparticles. The authors evaluated the antioxidant capacity of active chitosan films (30% flour or microparticles) on fresh walnuts, compared with walnuts packed in commercial (PE) and control chitosan bags. The results showed that in 31 days of testing, chitosan films enriched with olive by-products (flour or microparticles) exerted a greater protective effect against peroxides formation (1.78 ± 0.34 and 1.65 ± 0.14 mEq/kg for microparticles and flour olive pomace, respectively) compared to commercial (5.09 ± 0.14 mEq/kg) and control chitosan (3.10 ± 0.03 mEq/kg) films. The authors decided to evaluate the fatty acid profile (ω3, ω6 and ω3/ω6) of the walnuts during the same observation period and found that in both chitosan films (control and active) there were no significant changes in ω3 and ω6 content. Otherwise, a significant reduction in ω3 content was recorded in the commercial PE film and in the open packaging, thus confirming the protective effects of the active films. In a well-articulated research, Moudache et al. [61] focused the attention on olive leaves and cake extracts, rich in phenolic compounds (oleuropein, luteolin and hydroxytyrosol). After selecting the best extraction method, the most interesting antioxidant extract (ethanol: water 70/30 olive leaf extract) was dispersed in an aqueous adhesive solution, which was used to develop two types of multilayer films, PE-based films (PE/PE) (1%, 2%, 3%, 5% and 10% of extract) and PE/paper films (PE/P) (1% and 2% of extract). The scavenger activity of the active films was evaluated by exposing the films to a gas rich in free radicals, and the best performance in terms of antioxidant activity was recorded for PE/PE (10% olive leaf extract).

Recently, the extracts of olive leaves and pomace have also been applied to improve the properties of coating films. Khalifa et al. [62] used leaves or pomace extract (1% and 2%) to formulate active chitosan coatings and then compared these with a simple chitosan coating and a water wax coating incorporating thiabendazole. These coatings were sprayed on strawberries, fruit selected for its rapid kinetic decay. The authors observed that during 16 days of refrigerated storage the total phenolic content rapidly decreased in the uncoated products compared to fruits with active coating (the best effect with 2% of leaves extract). These results were also confirmed in a subsequent study of the same authors [63], who applied similar active coating to apples, monitoring the effectiveness during 35 days of storage at 4 °C. Moreover, in this case, the olive leaf extract significantly reduced the loss of total phenols and flavonoids in the apples (the best result with 2% olive leaves extract). Licciardello et al. [64], further than studying the antioxidant efficacy of by-product extracts, studied the release kinetics of extracts from active packaging films in three different food simulating systems, water, 10% ethanol and 50% ethanol. For this aim, shellac and cellulose nitrate films were coated with two active compounds, olive leaf extract and grape pomace extract. The study confirmed the antioxidant capacity of the two extracts and highlighted that release is strongly dependent on the solvent in contact with the film.

## 4. Fruit and Vegetable By-Products to Enhance Antimicrobial Properties of Polymeric Film

Most bioactive compounds extracted from by-products are able to affect microbial and fungal growth. Table 2 summarizes fruit and vegetable by-products adopted as peel or skin powder and as peel, skin or leaf extract to develop films with antimicrobial properties, tested against spoilage and pathogenic microorganisms. The finding of various researchers that utilized fruit by-products to develop active polymeric films are presented in the subsequent paragraph.

### Pomegranate, Grape and Grapefruit By-Products

As regard pomegranate by-products, the antimicrobial activity is linked to the high content of tannins, able to modify the morphology of the microbial membrane, thus causing cell lysis. In a recent study, Hanani et al. [67] confirmed the antimicrobial efficacy of pomegranate peel. Through the inhibition zone method, the authors observed that the active fish gelatin film with 5% pomegranate extract exhibited the highest efficacy against *Staphylococcus aureus* (inhibition zone of 7.0 mm), and other Gram-positive bacteria, as *Listeria monocytogenes*, (inhibition zone of 5.13 mm). On the other hand, Gram-negative bacteria as *Escherichia coli*, were found more resistant. This finding was in agreement with the study of Ali et al. [98] who investigated antimicrobial properties of starch-based films containing pomegranate peel powder (0%, 2%, 4%, 6%, 8%, 10%, 12%, and 14% w). The active films were found more effective against *S. aureus* than *Salmonella*. A similar study was carried out by Mushtaq et al. [97] who evaluated the antimicrobial effect of zein active packaging with pomegranate peel (25, 50 and 75 mg/g of film forming solution) against various pathogenic bacteria (*E. coli, P. perfringens, M. luteus, E. faecalis, S. aureus, P. vulgaris* and *S. typhii*). These authors found that active zein films exhibited great inhibitory zones against any tested strains, but the effect was dose dependent. The authors then applied the active zein packaging to Himalayan cheese (Kalari) for 30 days. The results showed inhibitory effects against total mesophilic bacteria, yeasts and molds and an action on delay of oxidative reactions of lipids. Many other investigations revealed antimicrobial potential of bio-based active packaging for shelf life extension of different foods. In particular, Emam-Djomeh et al. [93] confirmed that the effect of pomegranate peel extract (PPE 1, 1.5, and 2%) in casein-based film was more pronounced against *S. aureus* compared to *E. coli*. The authors also applied the active films as coating for meat samples (with 2% of PPE) and monitored the microbial growth (total mesophilic bacteria and *S. aureus*) for 12 days. Unfortunately, the authors did not record desired results, since the film exhibited reduced efficacy against *S. aureus*, most probably due to interactions with other microbial groups, such as *Pseudomonas* spp. Pomegranate peel extract has been also used to enhance the quality of shrimps during cold storage. Seafood is particularly sensitive to microbial and oxidative spoilage, because of high content in proteins, amino acids or omega-3 fatty acids, that generally cause changes in color, odor and texture. For these reasons, many synthetic preservatives can be applied, in particular sulphur dioxide and sulphites, effective to reduce microbial growth, oxidation and browning developing. Yuan et al. [94] proposed chitosan active coating with pomegranate peel extract (PPE 1.5%) on Pacific white shrimp, stored for 10 days under ice conditions. The results showed that active chitosan kept microbial growth lower than the control samples due to the antimicrobial capacity exhibited by both PPE and chitosan that induced cell lysis or cause other interference with intracellular components, both for the phenolic compounds of the extract and for the ability of chitosan to interact with the negatively charged microbial cell membrane. These results were also recently confirmed by the study of Licciardello et al. [95]. The authors observed that the incorporation of pomegranate peel extract (0.361 g/mL) into edible coatings based on chitosan and locust bean gum was promising, due to the synergistic effect shown by the active compounds. This coating, tested on shrimps, was able to reduce *Pseudomonas* spp. by 2 log units and to keep the psychrotrophic microbial load below the limit of 7 log cfu/g during 6 days of storage. Otherwise, Kharchoufi et al. [96] incorporated pomegranate peel extract (extracted with water—WPPE or methanol—MPPE) into two edible coatings, chitosan and locust bean gum. The authors studied the effect on growth of *Penicillium digitatum*, responsible for orange decay, compared to the biocontrol agent *Wickerhamomyces anomalus* or their combination. Experimental results showed that the addition of the extract in chitosan and locust bean gum significantly reduced green mold by 49% and 28%, respectively, compared to the relative controls.

Recently, Sogut and Seydim [84] placed chicken breast fillets between two layers of active chitosan film loaded with grape seed extract (GSE) at 5%, 10% and 15%, vacuum packed in LDPE and monitored their effectiveness on total mesophilic bacteria and coliforms for 15 days of storage. The preliminary antimicrobial efficacy of GSE on *L. monocytogenes*, *S. aureus, E. coli* and *P. aeruginosa* confirmed the greater sensitivity of Gram-positive bacteria, also found in previous applications with other extracts. The active film with 15% of extract kept the microbial proliferation below the acceptability limit for the entire storage period whereas in the control film, the meat became unacceptable within a few days. Alves et al. [85] observed a shelf-life prolongation of refrigerated salmon from 4 to 7 days of storage, applying on salmon surface a chitosan film with GSE and microcapsules of carvacrol. Active film efficacy was monitored for 14 days and compared to chitosan alone and to sample without film. The authors assessed that samples with active film controlled the total volatile basic nitrogen and the pH better than the control fish, thus promoting a shelf life prolongation.

Various studies deal with application of grapefruit seed extract (GFSE) to develop antimicrobial packaging films. In particular, Kanmani and Rhim [87,88] incorporated GFSE (0.6–13.3 μg/mL) in agar-based films and subsequently in carrageenan biopolymer. The antimicrobial activity showed large inhibitory zone against *Listeria monocytogenes*, with the maximum inhibition with the agar film containing 13.3 μg/mL (23.6 mm), while Gram-negative *E. coli* and *Bacillus cereus* were less susceptible, with an inhibitory zone of 5.66 and 8.66 mm, respectively. These findings were confirmed in carrageenan/GFSE film, where Gram-positive bacteria (*L. monocytogenes* and *S. aureus*) were more sensible than Gram-negative bacteria (*E. coli* and *B. cereus*), probably due to the additional outer membrane of the cell wall of Gram-negative bacteria. Wang and Rhim [91] noted that the GFSE efficacy can vary depending on the polymer matrix. In fact, these authors incorporated the GFSE in thermo-plasticized starch (TPS) as carrier, into plastic films made up of low-density polyethylene (LDPE) or PLA. The authors observed that while the PLA-TPS film exerted strong antibacterial activity against *E. coli* and *L. monocytogenes*, the LDPE-TPS exhibited lower effects. This result is due to the fact that GFSE was entrapped more tightly in the LDPE network than in PLA, thus suggesting that GFSE was released faster from PLA than LDPE. More recently, Shankar and Rhim [86] developed a synthetic polymer-coated paper (alginate, carboxymethyl cellulose and carrageenan) with GFSE (6.7 wt%). The biopolymer coating material (1, 2, 4 mg/mL) was tested on *L. monocytogenes* and *E. coli*. During 15 h of monitoring, the lowest concentration of coating material (1 mg/mL) exhibited a bacteriostatic activity against *E. coli*, while a bactericidal effect was found against *L. monocytogenes*. Furthermore, the highest concentration of coating material (4 mg/mL) stopped the viability of *L. monocytogens* immediately after 3 h, while for more resistant *E. coli* 9 h were needed. On the other hand, Kim et al. [89] noted that the layer-by-layer coating made with chitosan and alginate, both containing 0.5% of GFSE, was effective on shrimps stored for 15 days under refrigeration (4 °C). In fact, the total mesophilic bacteria were kept below the limit of 7 log cfu/g for 9 days, and the total psychrophilic bacteria exceeded the limit after 12 days, compared to uncoated samples that exceeded the limit after 6 days. The authors were able to state that for coated samples (chitosan-alginate-GFSE) an extended shelf life and a reduced production of total volatile nitrogen were recorded.

## 5. Fruit and Vegetable By-Products to Improve Physical and Mechanical Properties of Films

### 5.1. Fruit By-Products

The requirements of films and coatings in terms of physical and mechanical properties depend on characteristics of the food that is to be protected. Therefore, components such as crosslinkers and nano-reinforcements, to be added to the polymeric matrix to improve barrier, tensile and water resistance properties are of striking importance. As can be seen from Table 3, fruit by-products are abundantly investigated for this aim. In particular, Priyadarshi et al. [82] asserted that the incorporation of apricot kernel essential oil into chitosan film improved both physical and chemical properties. The authors observed that formation of covalent bonds between chitosan and functional groups of apricot kernel oil and the creation of a bilayer (chitosan/apricot kernel oil) reduced moisture content and water absorption of the active film compared to pure chitosan. Furthermore, the addition of apricot kernel oil improved the mechanical properties (increase in tensile strength of about 94%), reduced film solubility in water and improved the water vapor barrier properties (approximately 41%), due to the hydrophobic components in the polymeric structure. 

A different application has been reported by Luchese et al. [36], who developed cassava starch film, by compression molding, to valorize by-products from blueberry juice processing (powder 4, 8 and 12 wt%). The solubility of the active films decreased due to interactions between phenolic compounds and hydroxyl groups of starch and sorbitol, but the processing method made films more hydrophobic than the casting process. In addition, these active films showed an excellent barrier to UV light, due to the great opacity compared to the control.

Paper is a biodegradable material with moderate mechanical properties, but the high porosity and the very low barrier properties against moisture and gas limit its use. To improve the water vapor and gas properties of paper, Kasaai and Moosavi [99] extracted and used hydrophobic material from peel and leaf of mandarin to treat food-grade Kraft paper. The authors asserted that some fraction of extract occupied the space between the pores on the paper, other fraction created a thin layer on its surface. Therefore, the presence of hydrophobic material of the extracts, which have proven to be terpene hydrocarbons, allowed to significantly reduce the water vapor permeability, the air transmission rate and the peroxide value of treated paper, compared to control system. Even the antioxidant activity was increased with the increase of the extract, thus proving that citrus by-products not only improved the barrier properties but also the stability against oxidation. 

Shahbazi [51] observed that chitosan and gelatin films enriched with red grape seed extract (GSE) showed lower mechanical properties (tensile strength—TS, puncture force—PF and puncture deformation—PD) than ZEO-formulated films (*Ziziphora clinopodioides essential oil*). The author assumed that the interactions between phenolic compounds of GSE, chitosan and gelatin molecules influenced the microstructure of the film network (the distance between the chains), thus reducing the aforementioned properties. On the contrary, the presence of GSE and ZEO increased the hydrophobic interactions in polymeric matrix and improved the water vapor barrier properties of the active films compared to the control systems. Recently, Munir et al. [100] noted that both the nature of the extract and its final concentration may have different influence on film properties. In particular, pomegranate peel extract (2%, 4%, 6% PPE) improved tensile strength of Surimi-based edible films compared to GSE at the same concentrations. The different cross-linking ability of proteins and phenolic compounds justified the better water vapor barrier properties found in the film with 6% GSE. Mushtag et al. [97] noted that the films with pomegranate peel extract, having antimicrobial activity, also showed good mechanical properties with an increase in tensile strength. These results confirmed that the close interaction between the polymer matrix (zein molecules) and the phenolic compounds of the extract made the film more resistant to stress. In addition, the extract increased film water vapor barrier properties due to the presence of extract gallotannins, which are compounds able to connect the polymer chain by hydrophobic interactions.

By-products from mango processing industry are characterized by high content of polysaccharides, in particular in the peel, which represents about 7–24% of the whole fruit weight. Mango peel (powder 1.09% by weight) was used by Torres-Leon et al. [101] to formulate an edible film with and without addition of antioxidant extract from fruit kernel. The addition of mango kernel extract increased the film water vapor permeability, while the presence of carotenoids and anthocyanins in the mango peel conferred to the film good barrier to radiation in the light spectrum (600 nm). This active film was applied on peaches, thus showing 64% and 29% less production of ethylene and carbon dioxide and 39% less oxygen consumption compared to control fruit. Nor Adilah et al. [102] also used mango peel extract (1–5% MKE) into fish gelatin films. The incorporation of this extract (5%) slightly reduced the value of the water vapor permeability and the water solubility. Nawab et al. [103] evaluated the effect of guar and xanthan gums (10%, 20% and 30%) on the properties of mango kernel starch films. The mango kernel is a great source of starch, about 56% on dry basis. The authors observed that the solubility of the starch film worsened. The high affinity of guar and xanthan gums with water increased the solubility value from 37.69% to 44.63% and 41.12%, respectively. The hydrophilic nature of the two gums also increased the water vapor permeability compared to the control. In contrast, the oxygen barrier properties of the composite starch film (with 10% guar and xanthan gums) and the tensile strength (with 10% and 20% of gums) were improved compared to the control, due to the creation of more resistant network between starch and gums through hydrogen bonds.

### 5.2. Vegetable By-Products

Similarly to examples reported for fruit, Table 4 summarizes the effects of vegetable by-products on physical and mechanical properties of bio-based and composite films. Cellulosic fractions are a group of interesting compounds obtained from various vegetable by-products. Some cellulosic fractions have been extracted from uncommon plants. An example is represented by *Arundo donax*, which is a tall perennial cane; from its waste biomass, cellulosic fraction was extracted with different degrees of purification. The cellulosic fraction of this hemicellulose-rich waste was used to obtain highly porous and super-absorbent bio-based aerogels [104]. Aerogels were obtained by freeze-drying method, from aqueous suspensions of the fractions extracted from the waste, stems and leaves. All the tested aerogels had an excellent water and oil absorption capacity similar to chemically modified aerogels, due to the presence of hemicellulose that generally promotes water absorption. In addition, thanks to their proven antioxidant capacity, these systems could be used as bioactive pads for fresh red meat packaging against color loss and lipid oxidation.

Benito-Gonzales et al. [105] used cellulose fillers obtained by *Posidonia oceanica* waste biomass, to improve the starch films pre-conditioned with two different relative humidity (RH 85 and 53%). *Posidonia oceanica* is one of the most abundant aquatic plants, whose leaves are a natural source of lignocellulosic materials. The results of the study showed that the incorporation of cellulose significantly improved mechanical and water barrier properties. In particular, a maximum reduction of water vapor permeability of 54% was observed in samples with 40% of cellulose (when the pre-conditioned starch was at 53% RH) compared to pure starch. The best results were observed for films with pre-conditioned starch at 85% RH, because the addition of cellulose (5%, 10% and 20%) converted the hydrophilic nature of the film into the hydrophobic type. According to the authors, this change is mainly due to a better dispersion of the cellulose and the formation of stronger interactions between pre-conditioned starch and cellulose.

The most abundant source of cellulose among vegetable by-products is represented by the potato peel, in most cases used as animal feed. Recently, various applications of potato peel could be found in the literature. In particular, Xie et al. [106] developed a biodegradable film with potato peel (PP powder 3% and 5%), then adding curcumin (1–5%) for antioxidant properties and bacterial cellulose (BC 5, 10, 15%) as reinforcing agent. The PP+BC films exhibited an improvement in oxygen barrier properties and a decrease in transparency compared to the control. Furthermore, the addition of bacterial cellulose (BC 10%) improved the water vapor barrier properties by 35% and 30% in potato peel films (3% and 5%, respectively) and tensile strength. These results have been correlated to the interaction between PP and BC through hydrogen bonds but also to the dense network that developed with the presence of BC, preventing the transfer of humidity through the film. Ramesh and Radhakrishnam [77] used cellulose nanoparticles (2% CNP) obtained from potato peel waste, to develop three biopolymers with chitosan, PVA and their combination. The authors obtained a promising yield of cellulose nanoparticles (about 39.8%) using three chemical treatments (alkaline treatment, bleaching and acid hydrolysis). The integration of cellulose nanoparticles improved the flexibility and elasticity of CNP-PVA (tensile strength of 4.667 N/mm^2^ and elongation 140%) compared to PVA film (tensile strength of 3.121 N/mm^2^ and elongation 59.3%). Furthermore, cellulose nanoparticles allowed to increase molecular interactions, thus also improving the oxygen barrier properties of the biodegradable films. Potato peel waste was also used as a filler by Sugumaran et al. [107] to develop a composite film with linear low-density polyethylene (LLDPE) and chemical compatibilizers, to improve the adhesion between filler and polymer. The authors observed that increasing the filler content from 10% to 40%, the water absorption increased in all the tested films, in the range of 0.2% to 0.9% in bio-LLDPE without compatibilizer, while by 0.2–0.8% in bio-LLDPE with compatibilizers, compared to clean LLDPE with value < 0.02%. In addition, they asserted that the compatibilizers improved the tensile strength of bio-LLDPE, as the increase in potato peel from 10% to 40% reduced the tensile strength from 15% to 40% compared to clean LLDPE.

Zhao and Saldaña [76] used potato peel and cull (0.5, 1 and 1.3 g peel/g cull) with and without gallic acid. The addition of gallic acid worsened the water vapor barrier properties, because the hydroxyl groups present in gallic acid increased the affinity of film with water. As regard the mechanical properties, the tensile strength increased with the increase of potato peel content, due to the high fiber content (27–55% of cellulose, 11% hemicellulose and 7–14% lignin) compared to the cull which is instead rich in starch (80%). In order to improve the particle distribution of the two by-products in the film matrix, Borah et al. [108] used ultrasound (US) treatment on the film forming solution made up of potato peel (PP) and sweet lime pomace (SLP). The US treatment improved some mechanical properties such as film strength and elongation capacity. In addition, although the US treatment improved the water vapor permeability, the addition of lime pomace in PP has made a further improvement of this property. Furthermore, the composite film showed low solubility, thus confirming that ultrasound improved the structure of the film, making it more uniform and compact to hinder the passage of the water molecules. According to the literature, the incorporation of nanofibers extracted from renewable agro-industrial by-products could be a solution to improve physical and mechanical properties of biopolymers. An example is given in the study conducted by González et al. [109]. The large number of amino acids in the structure of soy protein isolate (SPI) confers hydrophilicity to this biopolymer, therefore the authors thought of reinforcing soy protein film with different quantities of cellulose nanofibers (CNF 10, 20 and 40%) obtained from soybean hulls and pods. The reinforcement increased film hydrophobicity with a reduction in swelling index (water adsorption capacity) from 1200% (control SPI without nanofibers) to 200% (SPI-CNF 40%). In addition, the CNF improved the mechanical properties by giving films with great rigidity and breaking strength. The water vapor barrier properties did not change significantly.

Starch-based foams are known to have poor mechanical properties and hydrophilic characteristics compared to petroleum-based packaging. For these reason, Cruz-Tirado et al. [44] evaluated the use of various concentrations (0–40% of *w/w*) of lignocellulosic fibers from sugarcane bagasse (SB, cellulose content 40–50%) and asparagus peel (AP, cellulose content 22%) to improve the mechanical properties of biodegradable trays based on sweet potato starch. The lowest cellulose content of the asparagus fibers gave starch trays greater hygroscopicity than starch/SB trays, which at high concentration (from 20%) exerted a water absorption capacity (<34.4 g of water per 100 g of dry mass) lower than the control (55.4 g of water per 100 g of dry mass). In addition, the SB (5% to 40%) were well incorporated in the starch matrix, thus giving greater flexibility and hardness to trays than starch/AP trays, where high concentrations developed discontinuities in starch matrix.

## 6. Final Considerations

**Figure 3:**
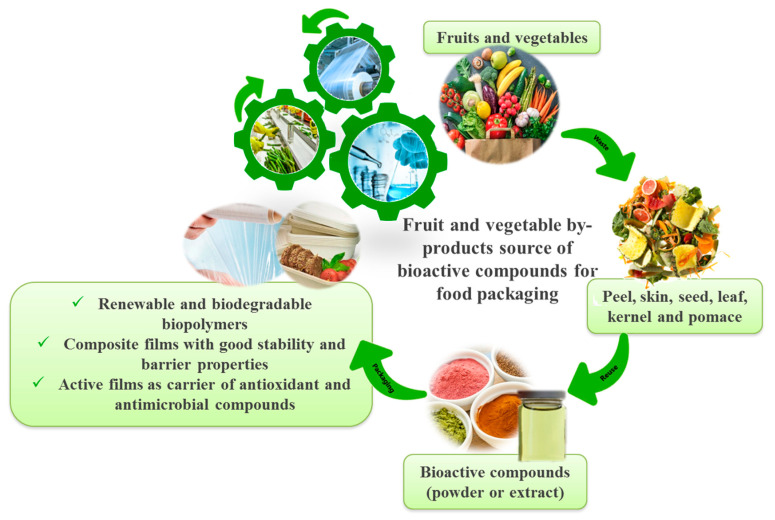
The potential application of agro-industrial by products to packaging materials.

In recent years, the interest in reusing materials of agro-industrial processes (peels, husks, pomace, seeds, etc.) increased significantly, due to the huge amount of these food residues, the need to face the problem of their disposal and the recognized value of by-products as source of bioactive compounds (polysaccharides, fibers, phenolic compounds, vitamins, minerals, etc.). It is also acknowledged that environmental impact generated by the petroleum-derived polymers paves the route for the development of greener alternatives and demands for replacing conventional plastics by renewable and biodegradable materials. In this context ideal candidates for green polymers should be the edible systems, i.e., materials with only food-grade components in their composition. Therefore, fruit and vegetable by-products could gain a great interest in the perspective of more sustainable packaging. The need for edible ingredients is not only limited to the film-forming matrix but also plasticizers and any other additive should be of natural origin. This overview demonstrates that scientific research is focused on this topic. Many fruit and vegetable by-products have been proposed to improve the properties of synthetic or bio-based plastic materials, which in many cases were also applied with success to real perishable products. The potential applications of agro-industrial residues to packaging materials discussed in this text, as well as the basic components and the remarkable characteristics of developed active systems, are illustrated in Figure 3. The picture highlights that findings recorded from these studies demonstrate obvious advantages in terms of food sustainability. Still, too few residues from the processing of fruit and vegetables are used appropriately. In order to use their potential effectively, more conscious efforts need to be made by modern sustainable food processing technology. The broad diffusion of knowledge from the scientific world to both food processing and packaging industrial sectors about the concrete advantages, the investment costs and the impact on the future state of the environment would really promote the valorization of fruit and vegetable by-products in plastic materials. 

## Figures and Tables

**Table 1:** Fruit and vegetable by-products and main phenolic compounds.

Products and By-Products Percentage		Main Phenolic Compounds	References
Apple (25–30% solid waste)	Pomace	Hydroxycinnamates, phloretin glycosides, quercetin glycosides, catechins, procyanidins, epicatechin, chlorogenic acid, cryptochlorogenic acid.	[39,40,41]
Artichoke (66% solid waste)	Bracts, leaves and stems	Chlorogenic acid, luteolin-7-O-rutinoside, luteolin-7-O-glucoside and apigenin.	[42,43]
Asparagus (40–50% solid waste)	Peel	Dietary fiber, rutin, peroxidases, phenols, flavonoids, hydroxycinnamic acids and saponins.	[44,45]
Blueberry (20–30% solid waste)	Pomace	Anthocyanins, cinnamic acid derivatives and flavonol-glycosides.	[46,47]
Citrus (50% solid waste)	Peel	Eriocitrin, hesperidin, naringin.	[48,49]
Grape (20% solid waste)	Skin	Catechins, epicatechins, epigallocatechin, picatechin gallate.	[50,51,52,53,54]
	Pomace	Catechins, anthocyanins, stilbenes, flavonol glycosides.	
	Seeds	Procyanidins.	
Mango (45% solid waste)	Kernel and leaves	Gallates, gallotannins, gallic acid, ellagic acid, glucosides.	[55,56,57,58,59]
Olive (30–50% liquid waste)	Pomace, peel and vegetation water	Myricetin, ferulic, sinapic caffeic, gallic, ellagic, oleuropein and hydroxytyrosol derivatives.	[60,61,62,63,64]
Pomegranate (49% solid waste)	Peel and pomace	Penduncalin, punicalagin, caffeic acid, chlorogenic acid, ellagic acid, apigenin, quercitin and gallic acid.	[65,66,67,68]
Husk (15–20% solid waste)	Barley husk	Vanillin, gallic acid, p-coumaric acid, p-hydroxybenzaldehyde, ferulic acid, syringic acid, p-hydroxibenzoic acid, vanillic acid and acetovanillon.	[69,70]
Onion (17% solid waste)	Skin	Quercetin 3,40-O-diglucoside and quercetin 40-O-monoglucoside and isorhamnetin-3-glucoside.	[71,72]
Peanut (35–40% solid waste)	Shell	Proanthocyanidins and procyanidins.	[73,74]
Potato (15% solid waste)	Peel	Chlorogenic acid, ferulic, gallic, protocatechuic and caffeic acid.	[75,76,77]
Tomato (20% solid waste)	Skin and seeds	Lycopene; Caffeic acid-glucoside, isomer I and isomer II, 3-caffeoylquinic acid, 5-caffeoylquinic acid, quercetin-triglucoside, quercetin-3-rutinoside, 3,4-di-O-caffeoylquinic acid, 3,4,5-tri-caffeoylquinic acid, naringenin chalcone and naringenin.	[78,79]

**Table 2:** Antimicrobial packaging with fruit and vegetable by-products.

By-Products	Active Packaging	Target Microorganisms	References
Apple skin	Chitosan edible film	*E. coli*, *S. enterica* and *L. monocytogenes*	[80]
Apple skin (powder, extract)	Composite films ASP/CMC	*L. monocytogenes*, *S. aureus*, *S. enterica and S. flexner*	[81]
Apricot kernel extract	Chitosan film	*E. coli* and *Bacillus subtilis*	[82]
Blueberry leaf extract	Chitosan coating	*S. aureus*, *L. monocytogenes*, *S. typhimurium*, *E. coli* and fungi	[83]
Grape seed extract	Chitosan film	*E. coli*, *L. monocytogenes*, *S. aureus* and *P. aeruginosa*	[84]
	Chitosan film and carvacrol	Mesophilic and psychrophilic bacteria and *Pseudomonas spp*	[85]
Grapefruit seed extract	Coated wrapping paper	*L. monocytogenes* and *E. coli*	[86]
	Biopolymer carrageenan	Gram-positive and Gram-negative food-borne pathogens	[87,88]
	Layer-by-layer coating with alginate, chitosan	Mesophilic and psychrotrophic bacteria	[89]
	Chitosan films	*Fungi*	[90]
	LDPE and PLA	*E. coli* and *L. monocytogenes*	[91]
Olive oil leaves and olive pomace	Chitosan film	*R. stolonifer*, *P. expansum*	[62,92]
Pomegranate peel extract	Casein-based film	*S. aureus*, *E. coli*	[93]
	Chitosan coating	Total aerobic bacteria, *Pseudomonas spp.*, *P. digitatum*	[94,95,96]
	Zein-based film	*E. coli*, *P. perfringens*, *M. luteus*, *E. faecalis*, *S. aureus*, *P. vulgaris* and *S. typhii*	[97]
Pomegranate peel powder	Fish Gelatin film	*S. aureus*, *L. monocytogenes* and *E. coli*	[67]
	Starch-based film	*S. aureus*, *Salmonella*	[98]

**Table 3:** Fruit by-products and their effects on physical and mechanical properties.

By-Products	Packaging System	Physical and Mechanical Properties	References
Apricot kernel oil (AKo)	Chitosan film with AKo (1:0, 1:0.125, 1:0.25, 1:0.5 and 1:1 *w/v*).	Essential oil improved TS* and WVB**, and reduced film solubility (from 18.42 to 4.76%).	[82]
Blueberry waste (BW)	Cassava starch film with BW powder (4, 8 and 12 wt%).	BW decreased SI** (pH 2.5, 7.0 and 10.0) and promoted UV protection.	[36]
Citrus peel and leaves	Kraft paper + peel:leaf extract (2:0, 2:1, 3:0).	Peel:leaf extract (2:1) increased WVB** and O_2_B**.	[99]
Grape seed (GSE)	Chitosan and gelatin films with GSE (1% *v/w*) and *Ziziphora clinopodioides* essential oil (ZEO).	1% GSE + 1% ZEO decreased TS*, PF*, PD* and SI**; increased WVB**.	[51]
Grape seed (GSE) + Pomegranate peel (PPE)	Surimi edible films with GSE + PPE (0%, 2%, 4% and 6%).	6% PPE improved TS*; 6% GSE increased WVB** and both reduced light transmission.	[100]
Mango peel and kernel (MKE)	Edible mango peel coating with MKE (0.078 g/L).	MKE reduced WVB** and film solubility (from 60.24 to 52.56%).	[101]
Mango peel extract (MPE)	Fish gelatin film with MPE (1%, 3% and 5%).	MPE improved TS* (from 7.65 to 15.78 MPa) and reduced solubility from 40% to 20%.	[102]
Mango kernel starch	Composite film (kernel starch and guar/xanthan gum 10%, 20% and 30%).	The different % of gums increased TS and O_2_B**, but decreased the film solubility and WVB*.	[103]
Pomegranate peel extract (PPE)	Zein film with PPE (0, 25, 50, and 75 mg/mL of film forming solution).	PPE improved TS* and WVB**, increased film solubility from 6.166% (control) to 18.29% (75 mg PPE).	[97]

* Mechanical properties: tensile strength (TS); puncture force (PF); puncture deformation (PD); ** physical properties: swelling index (SI); water vapor barrier (WVB); oxygen and carbon dioxide barrier (O_2_B and CO_2_B).

**Table 4:** Vegetable by-products and effects on physical and mechanical properties of films.

By-Products	Packaging System	Physical and Mechanical Properties	References
*Arundo donax*	Cellulose-based aerogels	Porous aerogel has great adsorption capacity.	[104]
*Posidonia oceanica*	Corn starch films with cellulose fillers	The stronger interaction between starch and cellulose improved TS* and WVB**.	[105]
Potato peel (PP)	PP film + Bacterial cellulose (BC) + curcumin	BC-10% improved TS*, WVB** and O_2_B**, while BC-15% reduced O_2_B** and WVB**.	[106]
	Cellulose nanoparticles in chitosan and/or PVA	Nanoparticles improved the flexibility, elasticity and O_2_B** of films.	[77]
	PP as filler (0–40%) in LLDPE	PP from 10% to 40% increased the water absorption and reduced TS* compared to the control.	[107]
Potato peel (PP) and cull	PP:cull ratio (g/g) (0:1, 0.5:1, 1:1, 1.3:1)	The ratio 1.3:1 improved film TS* and WVB** due to high fiber content of PP compared to cull.	[76]
Potato peel (PP) and sweet lime pomace (SLP)	Biopolymer films with PP and SLP	US-treatment improved WVB both in PP and in SLP films and reduced solubility.	[108]
Soybean hulls and pods	Cellulose nanofibers in soy protein films	Nano-fibers improved TS* and SI** of films.	[109]
Sugarcane bagasse (SB) and asparagus peel (AP)	Trays based on potato starch and fibers from SB or AP	Fibers improved thermal stability; SB make the trays less porous with higher TS* and lower SI** compared to control trays.	[44]

* mechanical properties: tensile strength (TS); puncture force (PF); Puncture deformation (PD); ** physical properties: swelling index (SI); water vapor barrier (WVB); oxygen and carbon dioxide barrier (O_2_B and CO_2_B).
